# Quantitative vs. Qualitative Outcomes: A Longitudinal Study of Risk and Ambiguity in Monetary and Medical Decision-Making

**DOI:** 10.21203/rs.3.rs-4249490/v1

**Published:** 2024-06-24

**Authors:** Chelsea Y. Xu, Ohad Dan, Ruonan Jia, Emily Wertheimer, Megha Chawla, Galit Fuhrmann-Alpert, Terri Fried, Ifat Levy

**Affiliations:** 1. Department of Comparative Medicine, Yale School of Medicine;; 2. Interdepartmental Neuroscience Program, Yale University;; 3. Department of Software and Information Systems Engineering, Ben Gurion University of the Negev;; 4. Department of Internal Medicine, Yale School of Medicine;; 5 Department of Neuroscience, Yale School of Medicine;; 6. Department of Psychology, Yale University;; 7. Wu-Tsai Institute, Yale University

**Keywords:** Decision-making, risk and ambiguity, medical decisions, qualitative outcomes

## Abstract

How do decision-makers choose between alternatives offering outcomes that are not easily quantifiable? Previous literature on decisions under uncertainty focused on alternatives with quantifiable outcomes, for example monetary lotteries. In such scenarios, decision-makers make decisions based on success chance, outcome magnitude, and individual preferences for uncertainty. It is not clear, however, how individuals construct subjective values when outcomes are not directly quantifiable. To explore how decision-makers choose when facing non-quantifiable outcomes, we focus here on medical decisions with qualitative outcomes. Specifically, we ask whether decision-makers exhibit the same attitudes towards two types of uncertainty - risk and ambiguity - across domains with quantitative and qualitative outcomes. To answer this question, we designed an online decision-making task where participants made binary choices between alternatives offering either guaranteed lower outcomes or potentially higher outcomes that are associated with some risk and ambiguity. The outcomes of choices were either different magnitudes of monetary gains or levels of improvement in a medical condition. We recruited 429 online participants and repeated the survey in two waves, which allowed us to compare the between-domain attitude consistency with within-domain consistency, over time. We found that risk and ambiguity attitudes were moderately correlated across domains. Over time, risk attitudes had slightly higher correlations compared to across domains, while in ambiguity over-time correlations were slightly weaker. These findings are consistent with the conceptualization of risk attitude as more trait-like, and ambiguity attitudes as more state-like. We discuss the implications and applicability of our novel modeling approach to broader contexts with non-quantifiable outcomes.

## Introduction

Decision-makers are often required to choose between outcomes that are not quantifiable but can still be easily ranked by their goodness. Consider for example an individual with a mobility impairment, facing a choice between two treatments. The first treatment will allow the patient to walk with some assistance, a moderate improvement to their condition; the second treatment will lead to a complete recovery. Clearly, a guaranteed recovery is better than a guaranteed moderate improvement. However, in many cases, outcomes are not guaranteed but rather are associated with some level of uncertainty. In such scenarios, it is not clear how to integrate qualitative outcomes with the level of uncertainty surrounding them. How do humans make choices in such cases?

Compared to such qualitative outcomes, decisions between uncertain quantifiable outcomes are more straightforward and have been extensively studied. A core concept that binds quantitative outcomes with their likelihoods is the objective measure of expected value (Schoemaker, 1982). For example, the expected value of a lottery offering $10 with a 50% chance is $5. In contrast to this objective value, to take into account individual preferences, decision theories often use subjective values (Fischhoff et al., 1983). For example, a decision-maker who is averse to uncertainty would generally prefer a certain $5 over a 50% chance of receiving $10, implying that for the decision-maker, the subjective value of the lottery is lower than that of $5.

To incorporate individual uncertainty preferences into the subjective valuation, previous literature distinguished between two types of uncertainty: risk and ambiguity (Ellsberg, 1961; I. Levy et al., 2010). Risk describes a fully known probability, for example, a lottery with a 50% winning chance. Ambiguity may arise from several factors (Dan et al., 2024) and describes uncertainty that is not precisely known, for example, a lottery with 25% to 75% winning chance.

Previous research on uncertainty attitudes focused mainly on monetary outcomes, finding individual variability in uncertainty attitudes (Borghans et al., 2009; Tymula et al., 2013), with a general aversion to both risk and ambiguity (Dicks & Fulghieri, 2019; Kimball, 1993; I. Levy et al., 2010; Machina & Siniscalchi, 2014). Similar findings were found in studies that used non-monetary, but still quantifiable outcomes such as the number of M&M’s (D. J. Levy & Glimcher, 2011) or dosage of medication (Seaman et al., 2016). In contrast to these quantifiable outcomes, many real-life decisions include non-quantifiable outcomes, such as future activities, social consequences, or, as in the example above, medical hazards and benefits (Shiffrin, 2022). To investigate how decision-makers behave in contexts implicating unquantifiable outcomes, here we focus on medical decision-making.

Prior research has shown that decision-makers incorporate outcome probabilities into their medical choices (Fried et al., 2002) as well as the ambiguity around these probabilities (Han et al., 2007, 2011), and individual risk perception (Brewer et al., 2007; Fried et al., 2002). Medical decision-making studies can include quantifiable outcomes, for example, the number of months added to a patient’s life expectancy (Attema et al., 2018). However, in ecological settings, when outcomes are often qualitative, it remains unclear how different outcome levels are integrated with risk and ambiguity. More broadly, it is not clear whether uncertainty attitudes that individuals apply in quantitative domains are translatable to decisions involving non-quantitative outcomes.

**Figure 1.**
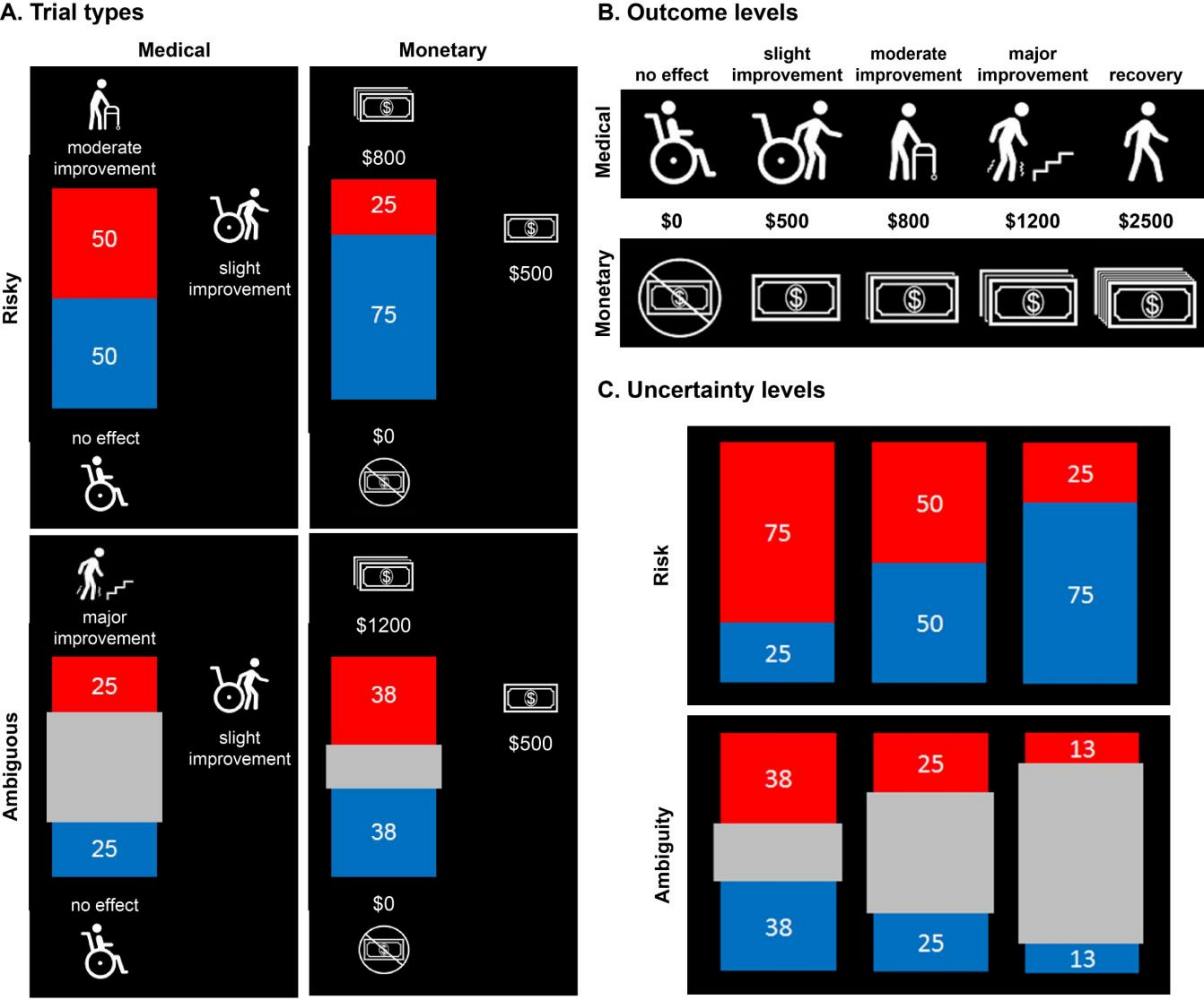
Study design. **A**: Task design and four decision scenarios: participants chose between an uncertain option and a sure outcome under four conditions: risk medical, ambiguous medical, risky monetary, and ambiguous monetary. **B**: All possible outcomes in the medical and monetary scenarios. **C**: Levels of probability (75%, 50%, and 25%) and ambiguity (24%, 50%, and 74%) for the uncertain option.

To answer this question, we designed a decision-making task including both monetary and medical outcomes (Figure 1), and administered it online in two phases separated by a 9-month interval. This allowed us to compare the stability of uncertainty attitudes between domains with the stability of uncertainty attitudes over time within domain. Using both model-free and model-based analysis approaches, we show that risk and ambiguity attitudes in one domain significantly predict these attitudes in the other domain. We also show that both risk and ambiguity attitudes are moderately stable over time, with risk showing a slightly higher consistency. We find that this level of temporal stability is comparable to the consistency observed across different domains.

## Methods

### Participants, repeated measures, and exclusion criteria

Participants were enlisted through the Amazon Mechanical Turk (MTurk) online platform and redirected to a Qualtrics-based survey. Informed consent was obtained from all participants prior to their participation. Compensation for participation varied based on behavior in the experimental task, with amounts ranging from $4 to $10.

The study was conducted in two phases: an initial phase from March to May 2020 and a follow-up session approximately 9 months later, from January to February 2021. In the initial phase, 444 participants, ages 20–80 (mean ± standard deviation: 49.4 ± 14.8). After excluding data from 15 participants with duplicate worker identification numbers (MTurk ID), the remaining 429 participants were included in the analysis.

In the follow-up session, 306 participants (ages: 23–78, mean ± standard deviation: 49.1 ± 14.8) completed the study. Additional exclusion criteria in the second phase included a within-participant sequential age gap exceeding a 2-year threshold. As a result, 34 participants were excluded from the follow-up session. Finally, participants whose behavior indicated a lack of understanding of the task were excluded from the analysis (see below), resulting in an additional exclusion of 50 and 11 participants in the first and second phases respectively.

### Experimental task: Decision-making under uncertainty in medical and monetary domains

The decision-making task involved two separate hypothetical scenarios: one monetary and one medical (Fig.1). The experiment consisted of 98 trials evenly split between the two conditions. The monetary condition, based on previous studies (Grubb et al., 2016; Jia et al., 2023; D. J. Levy & Glimcher, 2011; Ruderman et al., 2016), required participants to choose between a fixed monetary gain of $500 and a lottery whose amount and probability of receipt varied. The amounts and probabilities were displayed numerically and graphically (Fig. 1B). The outcome probability of each lottery was represented by an image of a rectangle shaded red and blue. On each trial, one color (red or blue) was associated with monetary gain ($500, $800, $1200, or $2500), and the other was associated with the null outcome ($0). These pairings were randomized across trials. The size of the colored areas presented in the experiment (see Fig. 1) represented the probability of receiving the indicated outcome. Half of the trials involved risky decisions whose levels of risk were systematically manipulated (25%, 50%, 75%; “Risk” Fig. 1C). The other half of the trials involved ambiguous decisions where the outcome probabilities were only partially known. This was achieved by occluding the middle part of the colored image with a grey bar (“Ambiguity” Fig. 1C). The amount of the image covered varied (24%, 50%, 75%) thus creating three levels of ambiguity.

In the medical scenario, participants were asked to imagine that they were injured in a car accident. In this hypothetical scenario, the participant suffered a severe spinal injury that resulted in paralysis of their legs and, upon being rushed to the hospital, were told that their legs would remain paralyzed without medical intervention (see Supplementary Information). Mirroring the monetary condition, participants were called to choose between a conservative treatment that offered a certain but minor improvement in their condition (“slight improvement”) or an experimental treatment that offered uncertain but more substantial improvement. The uncertain outcomes value varied across several qualitative levels: no effect, slight improvement, moderate improvement, major improvement, or recovery. These probabilistic outcomes were represented verbally and graphically (Fig. 1B) and were explained to participants prior to the task (see Supplementary Information for descriptions of all medical outcome levels). Participants were assessed on their understanding of these pairings before starting the task. Like in the monetary condition, half of the trials in the medical condition were risky and half were ambiguous. The level of uncertainty in risky trials varied between 25%, 50%, and 75%. Similarly, ambiguity was induced by occluding the middle of the stimulus with a grey bar such that the level of uncertainty in these ambiguous trials varied between 24%, 50%, and 75%.

Since the medical outcomes were, by definition, hypothetical, we chose to use hypothetical outcomes for the monetary condition as well. In both conditions, all risk and ambiguity levels were paired with all outcome levels, and the color associated with the better outcome was randomized across trials. This resulted in 49 trials with a unique combination of uncertainty, outcome level, and color associated with positive outcomes in each domain.

For each domain, of these 49 total trials, 7 included a lottery whose payoff equaled the reference option (i.e., $500 in the monetary domain and slight improvement in the medical domain). Participants were excluded if they selected the uncertain reference option over the certain reference option more than 50% of the time. For the monetary domain, this resulted in 37 participants excluded from analysis in the first phase and 10 in the second. For the medical domain, this resulted in 33 participants excluded in the first phase and 4 in the second. As some of these participants had behavior excluded in both domains, in total 50 participants were excluded in the first phase and 11 in the second.

For the modeling analyses, in addition to these exclusions, participants were also excluded if they never selected an uncertain option, since these types of decisions cannot be modeled by our design. This resulted in an additional 53 (monetary) and 17 (medical) participants excluded in the first phase and 20 (monetary) and 6 (medical) excluded in the second phase.

### Model-free attitudes toward risk and ambiguity

To investigate the effect of outcome magnitude on choice behavior, we first calculated the proportion of trials in which the uncertain option was chosen over the reference option. For each participant in each domain, trials were collapsed across uncertainty levels and grouped by outcome level, so that the number of lottery choices was divided by the total number of trials for risky and ambiguous conditions in both the medical and monetary scenarios. To examine how uncertainty level influenced choice behavior, we calculated the proportion of trials in which the participant chose the uncertain option by grouping the uncertainty levels within each uncertainty type (i.e. risk, ambiguity) while collapsing across outcome levels. To estimate model-free attitude towards risk, we then calculated the mean choice proportion for all risk levels to generate a single value representative of risk preference for each participant.

For decisions involving risky options, the choice proportion directly reflects participant’s risk attitude. However, for decisions involving ambiguous options, the choice proportion reflects attitudes toward both risk and ambiguity. This is due to the fact that participants had to first estimate the outcome probability based on their ambiguity attitude and then make their choice based on their risk attitude. In the present design, a participant completely indifferent to ambiguity should act as if the outcome probability in all ambiguous trials is 50%. Thus, we subtracted the choice proportion of 50% risk trials from the choice proportion of trials within each level of ambiguity. This corrected choice proportion should reflect participants’ preference towards ambiguity with negative values indicating ambiguity aversion and positive values indicating ambiguity seeking. We then calculated the mean corrected choice proportion for all ambiguity levels to generate a single value representative of each participant’s attitude towards ambiguity.

### Model-based estimation of risk and ambiguity attitudes

We fit each participant’s choice data with models that are modified forms of a well-validated behavioral economics model used in previous studies (Jia et al., 2023; I. Levy et al., 2010; Ruderman et al., 2016; Tymula et al., 2013). We proposed two different models, and for each model, fitting was conducted separately for medical and monetary choices. All models separate the decision process into two steps: valuation and choice. They differ in the first valuation step but are the same in the choice step.

#### The valuation step:

The subjective value (SV) of each option is calculated by the two models as shown below.

##### Model 1 – Ordinal values:


(Eq. 1) $$SV=\left[P-\beta_{1}\left(\frac{A}{2}\right)\right]\times V^{\alpha_{1}}$$

where P is the outcome probability (0.25, 0.50, or 0.75 for risky lotteries, 0.5 for ambiguous lotteries and 1 for the certain option); A is the ambiguity level (0.24, 0.5, or 0.74 for ambiguous lotteries; 0 for risky lotteries and the certain option). This model is similar to the one used in Levy et al 2010, except that we used ordinal values to describe the monetary and medical outcomes, since medical outcomes do not have intrinsic cardinal values (monetary: 1 – $5000, 2 – $8000, 3 – $12000 and 4 – $25000; medical: 0 – no effect, 1 – slight improvement, 2 – moderate improvement, 3 – major improvement, 4 – recovery). Two parameters, α_1_ and β_1,_ are fitted for each participant representing risk and ambiguity attitudes respectively. Parameter α_1_ represents the risk attitude by the curvature of the utility function, with smaller α_1_ indicating greater risk aversion (or reduced risk seeking). Ambiguity attitude β_1_ is modeled by discounting the lottery probability linearly by the ambiguity level, weighted by β_1_. A participant is averse to ambiguity when β_1_ > 0, and is ambiguity seeking when β_1_ < 0 in both domains.

##### Model 2 – Rating:


(Eq. 2) $$SV=\left[P-\beta_{2}\left(\frac{A}{2}\right)\right]\times R^{\alpha_{2}}$$

where P and A are the same as in Model 1. The only difference from Model 1 is that Model 2 substitutes V for R, which is the self-reported rating of each outcome level in each domain. Thus, parameter α_2_ represents the curvature of the utility-rating function, which should be interpreted differently from risk attitudes (because the ratings may already incorporate the classical curvature of the utility function). Ambiguity attitude β_2_ is modeled and interpreted in the same way as β_1_.

#### The choice step:

The choice process is modeled in the same way, by a standard soft-max function,

(3) $$P_{\text{V}}=\frac{1}{1+{\text{e}}^{-\gamma \left(SV_{\text{L}}-SV_{\text{C}}\right)}}$$

where P_V_ is the lottery choice probability, SV_C_ and SV_L_ are the subjective values of the certain option and the lottery respectively, calculated by equation (1); γ is an individual-specific noise parameter.

Model-fitting was conducted in Matlab (Version R2021b) by maximum likelihood. We used Matlab optimizer fminsearch to minimize the negative log-likelihood function. Maximum likelihood was conducted by grid search, using different initial parameter values as the search starting points. Search starting points for α, β, and γ were within the range of [0.01, 4], [−2, 2], and [−1, −0.01] respectively, and grids were generated by a step of 0.5. For easier interpretation of data, we transformed the ambiguity attitudes in both domains as - β such that negative values indicate ambiguity aversion and positive values indicate ambiguity seeking.

Models were compared by both Bayesian Information Criteria (BIC) and cross-validation in Matlab (Version R2021b). Procedures of the model-fitting were the same as above.

##### BIC:

For each model-fitting in each domain and phase, we averaged the Bayesian information criterion (BIC) across all participants and compared it between the models and two decision domains.

##### 5-fold cross-validation:

For each participant in each domain, we randomly partitioned the data into 5 segments, each containing 9 or 10 trials. In each of the 5 iterations, we trained the model with 4 segments of the choice data, and then predicted the choices for the left-out segment. We then calculated the mean squared error (MSE) between the predicted choices and the actual choices in the left-out segment. We averaged the MSE across all 5 iterations for each participant. This process was repeated for each model, and the MSE was compared between 2 models by averaging across all participants. We selected the best model by these two criteria, and used model fitted parameters from the best model to interpret participants’ behavior.

## Results

We recruited 429 participants for a two-phase longitudinal online study, of whom 272 continued to partake in the second phase. The study included a decision-making task with monetary and medical outcomes in hypothetical scenarios (Methods, Fig. 1). Monetary scenarios included choices between a guaranteed $500, and a lottery offering potential greater outcomes. For example, a lottery providing $1,000 with 50% chance and $0 with 50% chance. Similar to these monetary decisions, participants made a series of medical decisions, choosing between a hypothetical treatment that guaranteed a minor improvement, and one that offered a more significant improvement with some chance. For example, a complete recovery with 50% chance and no improvement at all with 50% chance.

### Sensitivity to outcome level

**Figure 2.**
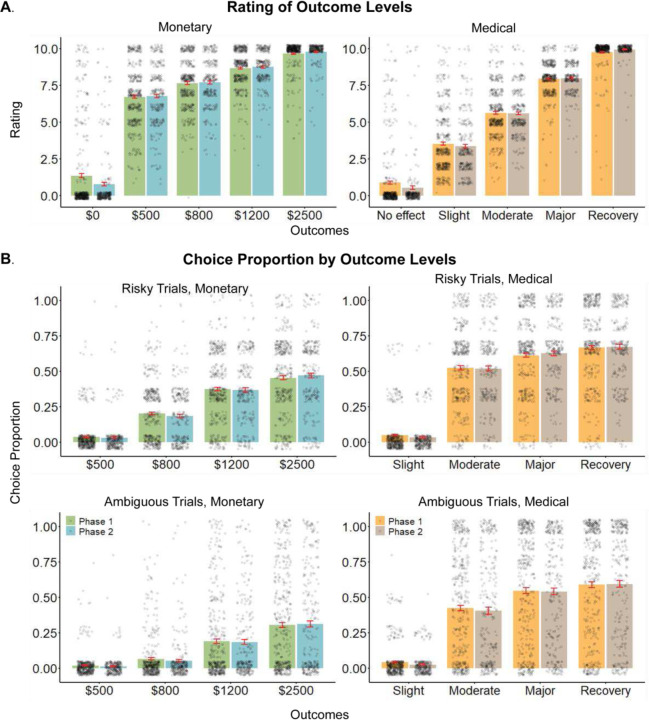
Ratings and choice proportion by outcome level. In both monetary (left) and medical (right) outcomes, participants demonstrated higher valuations for better outcomes in (**A**) subjective ratings and (**B**) choices. Choice proportions reflect the preference for the presented outcome over all risk and ambiguity levels (top and bottom), when contrasted with a certain low outcome. Grey dots are single participants. Bars and error bars are average and standard errors, measured separately in the 16 two phases of data collection.

In both the monetary and medical domains uncertain outcomes had several levels (Fig. 1). As a manipulation check, we verified that participants assigned greater subjective values to greater outcomes. To this end, we asked participants to rate on a 0–10 scale how pleasant they find each outcome. As expected, we found a monotonic relationship between outcome magnitude and its subjective pleasantness (Fig 2.A). As a second manipulation check, we observed the choices participants made in the decision-making task and calculated the overall proportion of choices in the probabilistic options per each outcome level. Here again, we found a monotonic relation between the proportion of choices in each outcome level and its magnitude (Fig. 2.B). These results suggest that participants comprehended the tasks and acted in ways that aimed to maximize their outcomes across both the monetary and medical scenarios.

### Risk and Ambiguity attitudes in the monetary and medical domains

**Figure 3.**
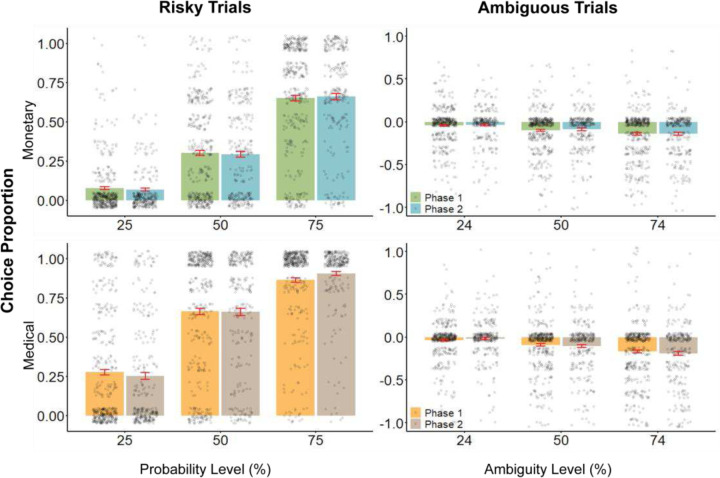
Choice proportion by uncertainty level. Left: Choice proportion of the risky options summarized by success probability in monetary and medical domains (top and bottom, respectively). Right: Proportion of choices in ambiguous options corrected by risk proportion (Methods). A corrected proportion of 0 represents ambiguity neutrality. Grey dots are individual participants, bars and error bars are mean and standard error.

Next, we assessed participants behavior in risky and ambiguous decisions. Collapsing across all outcome magnitudes, we calculated for each participant the proportion of trials in which they chose the risky option in each domain, grouped by uncertainty level (Fig. 3, left). In both the monetary and medical domains, participants increasingly opted for the riskier choice as the probability for a positive outcome rose, demonstrating sensitivity to expected outcomes.

Choices in ambiguous options are driven in part by risk attitudes and hence the proportion of choices in ambiguous options are, on their own, not as simply interpreted (Methods). Instead, to assess ambiguity attitudes we corrected for each participant the proportion of choices in ambiguous trials by their individual proportion of choices in risky trials (Methods). With this measure, a corrected proportion of 0 represents ambiguity neutrality and negative values indicate ambiguity aversion. In both domains, we found that greater levels of ambiguity were associated with more negative corrected scores, indicating a general aversion to ambiguity (Fig. 3, right).

### Uncertainty attitudes are correlated across domains

**Figure 4.**
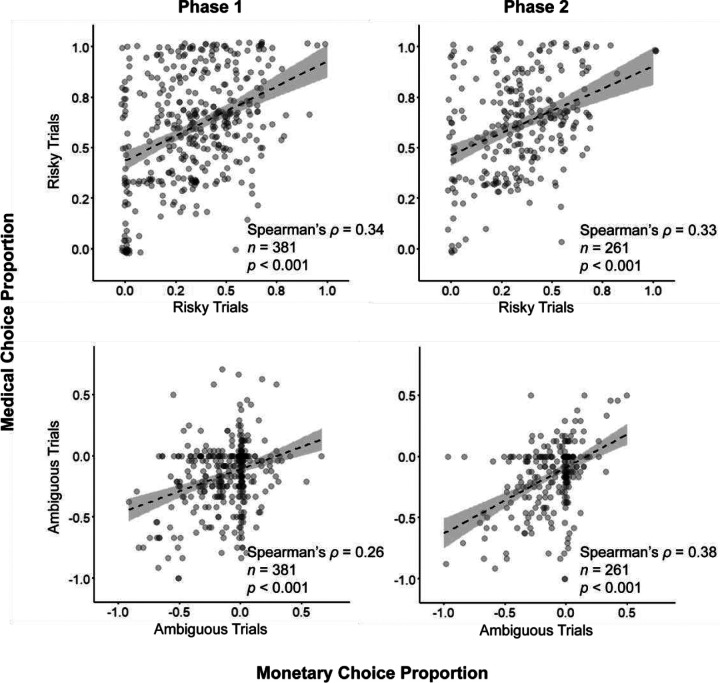
Correlation of uncertainty attitudes across domains. Grey points are the individual proportion of choices in risky trials (top) and the corrected proportion of choices in ambiguous trials (bottom), collected in timepoints 1 and 2 (left and right). Dashed line is linear fit of the data. Shaded area is 95% confidence interval.

Having summarized the risk and ambiguity attitudes over participants, we next asked whether these attitudes were correlated between the medical and monetary domains within participants. To answer this question, we calculated for each participant in each domain, the overall proportion of choices in all risky trials, and the corrected proportion of choices in all ambiguous trials. We found that the proportions of risky choices were moderately correlated across domains in the first data-collection phase, a finding that was replicated in the second phase (Spearman’s ρ = 0.34, and ρ = 0.33; in both *p* < 0.001; Fig. 4, top). Similarly, the corrected choice proportion in ambiguous trials was significantly correlated between the two domains in phase one and two (Spearman’s ρ = 0.26, and ρ = 0.38; both *p* < 0.001; Fig. 4, bottom).

### Uncertainty attitudes persistence over time

**Figure 5.**
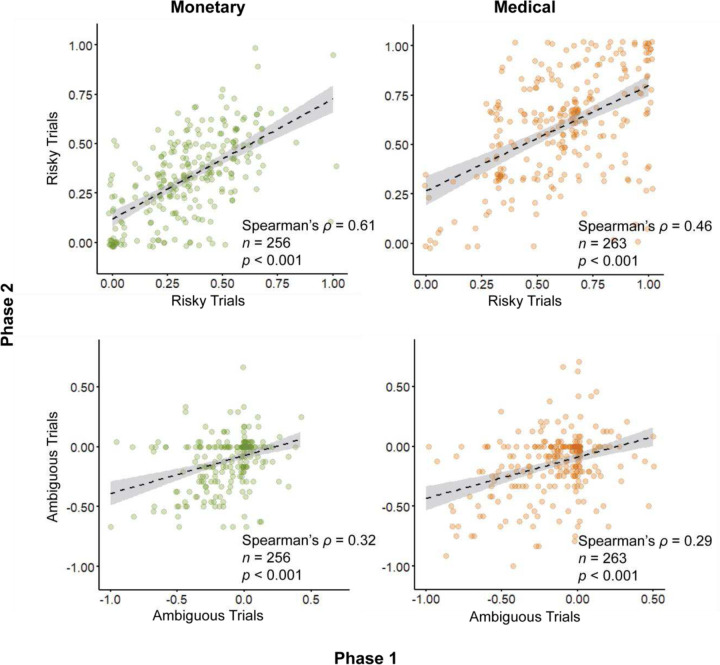
Uncertainty attitudes across time. Uncertainty attitudes were calculated for data collected in phase 1 (abscissa) and 9 months later in phase 2 (ordinate). Dots are proportions of choices in risky options (top) and corrected proportions of choices in ambiguous options (bottom) in monetary and medical domains (left and right) calculated each participant. Dashed line is linear fit of the data. Shaded area is 95% confidence interval.

We re-administered the decision-making task following a nine-month interval, allowing us to determine the stability of uncertainty attitudes over time. To assess individual risk and ambiguity attitudes we calculated for each participant the overall proportion of choices in risky options, and the corrected proportion of choices in ambiguous options. Considering the two time points, we find a high correlation in risk attitudes in the monetary domain, and a moderate correlation in the medical domain (Spearman’s ρ = 0.61, and ρ = 0.46 respectively; in both, *p* < 0.001; Fig. 5, top). In ambiguity, we found a moderate-low correlation in both the monetary and medical domains (Spearman’s ρ = 0.32 and ρ = 0.29 respectively; for both *p* < 0.001; Fig. 5, bottom).

### Model-based estimation of uncertainty attitudes

Our assessments of uncertainty attitudes were thus far based on the proportion of trials in which uncertain options were preferred over certain ones, regardless of the specific attributes of the chosen options. While useful, this approach cannot account for nuanced behavioral patterns (Methods). Next, we utilized a model-based approach that provides a more accurate estimation of risk and ambiguity attitudes. Specifically, we utilized a standard economic decision-making model that our group has used before (Jia et al., 2023; I. Levy et al., 2010; Ruderman et al., 2016).

At its core, the model transforms an objective expected value of a lottery into a subjective value by incorporating individual uncertainty attitudes (Methods). For example, the objective expected value of a lottery offering a 50% for receiving $10 is $5. To represent the subjective value of a risk averse (or seeking) participant, the model outputs a utility value that is lower (or higher) than that of a guaranteed $5.

To calculate the objective value of an option, the decision-making model first considers the outcome offered by the option, for example a specific dollar amount (Methods). While this is straightforward for a monetary - or any quantitative - outcome, it is not clear how to adapt the model to consider qualitative outcomes such as the medical improvement levels used in our paradigm. To accommodate such outcomes in the model, we tested two variations that substitute the qualitative medical outcomes with specific numerical values. The first, the Ordinal model, assigns an arbitrary ordinal value to the outcomes (e.g., slight improvement: 1, moderate improvement: 2, etc.; see Methods, Eq. 1). The second variation, the subjective Rating model, substitutes descriptive outcomes by explicit desirability ratings that participants provided for each of the outcomes (Methods, Eq. 2).

To arbitrate the two variations, we first tested the models’ performance in the more standard domain, monetary outcomes, using two criteria: cross-validation, and Bayesian information criterion (see Methods). Using both criteria, we found that the ordinal model was superior to the rating model (Supplementary). Hence, in subsequent analysis, we focused on the ordinal model.

**Figure 6.**
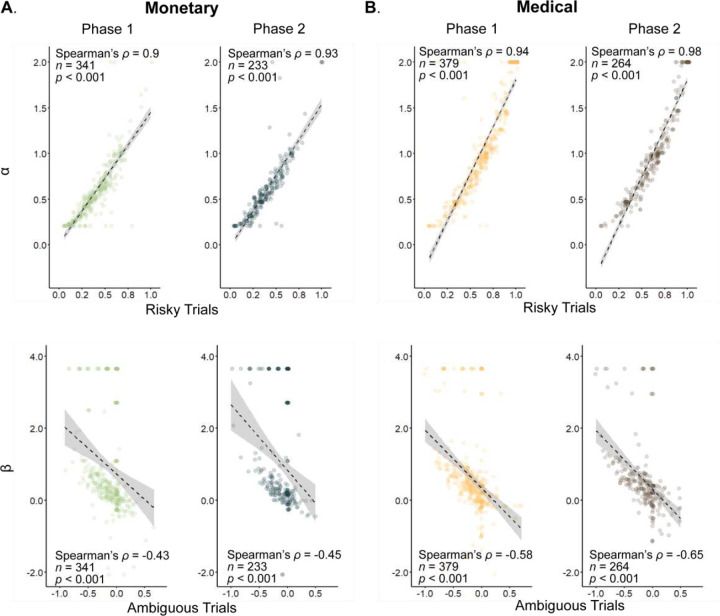
Correlations between uncertainty attitudes inferred from the model and from choice proportions. Each circle represents single-participant risk (top) and ambiguity (bottom) attitudes in the monetary and medical domains (**A**, and **B**). In all panels, each participant’s uncertainty attitudes are estimated from the model (ordinate) and choice proportion (abscissa). Dashed black lines and shaded areas are linear fit with 95% confidence interval.

Finding that the ordinal model has better predictive power compared to other model variations does not, on its own, mean that its predictive power is substantial. To confirm that the ordinal model fits the data well, we assessed the correlation between the uncertainty attitudes inferred from the model with those inferred from the choice proportions. We found that model-based risk attitudes (α in the model) were highly correlated with the risk attitudes assessed by the proportion of choices in risky options (phase one and two: Spearman’s ρ = 0.9, and ρ = 0.93, both *p* < 0.001; Fig 6A, top). Similarly, but with lower magnitude, we found a significant correlation between the model-calculated ambiguity attitude (β) and the corrected choice proportion in ambiguous trials (phase one and two: Spearman’s ρ = −0.43 and ρ = −0.45, both *p* < 0.001, Fig 6A, bottom).

Given the effectiveness of the ordinal model in the monetary domain, we next applied it to the medical domain and found similar correlations between the uncertainty attitudes inferred from the model and those observed in choice proportions. The correlation between risk attitudes inferred from the model (α) and from choice proportions was very high (phase one and two: Spearman’s ρ = 0.94 and ρ = 0.98; both *p* < 0.001; Fig. 6B, top). Similarly in ambiguity, we found high correlations between the model-based (β) and choice proportions (phase one and two: Spearman’s ρ = −0.58 and ρ = −0.65; both *p* < 0.001; Fig 6B, bottom). Together these results suggest that the ordinal model performs well in fitting the behavior both in the monetary and medical domains.

### Model-based uncertainty attitudes are correlated between domains and timepoints

Our model allows us to compare uncertainty attitudes across the medical and monetary domains. Additionally, our longitudinal study design uniquely offers a benchmark to compare the persistence of uncertainty attitudes between-domains, with the persistence of uncertainty attitudes within-domain, over time.

**Figure 7.**
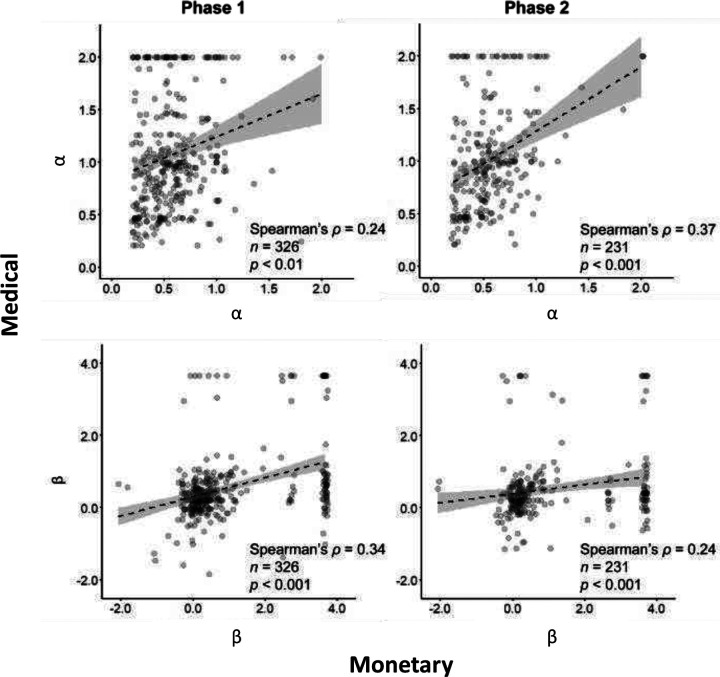
Across-domain correlations in model-based uncertainty attitudes. Each dot represents the model-based risk (top) and ambiguity (bottom) attitude of a single participant, estimated in the monetary (abscissa) and medical (ordinate) domains, in phase 1 (left) and phase 2 (right) of the study. Dashed black line and shaded area are linear fit with 95% confidence interval.

Across domains, we found low to moderate correlations between uncertainty attitudes (Fig. 7, bottom). Medical and monetary risk attitudes (α) were correlated in both time points (phase one: Spearman’s ρ = 0.24, *p* < 0.01; phase 2: Spearman’s ρ = 0.37, *p* < 0.001; Fig. 7, top). Similarly, ambiguity attitudes (β) had also moderate-low correlations across domain (phase one and two: Spearman’s ρ = 0.34 and ρ = 0.24; for both *p* < 0.001; Fig 7, bottom).

**Figure 8.**
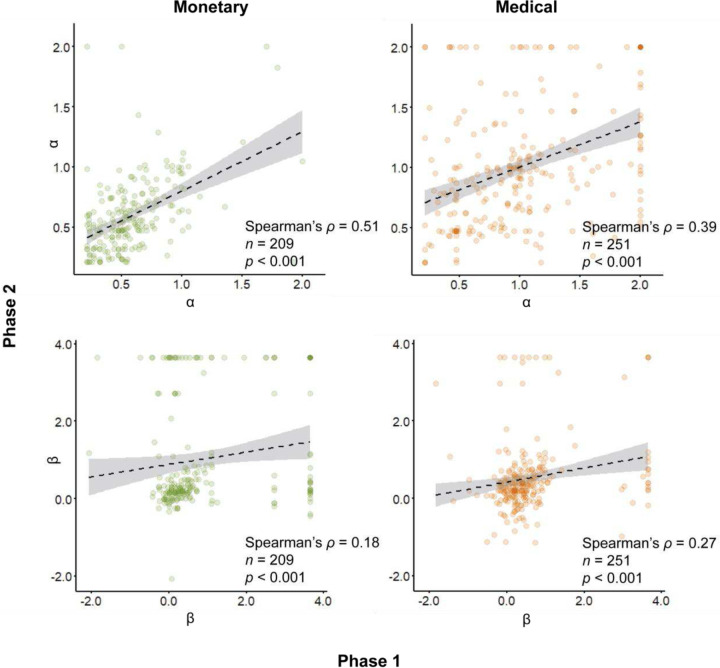
Across-time correlations in model-based uncertainty attitudes. Each dot represents the model-based risk (top) and ambiguity (bottom) attitude of a single participant, estimated in the monetary (left) and medical (right) domains, across the two study phases. Dashed black line and shaded area are linear fit with 95% confidence interval.

Next, we compared the cross-domain correlations with within-domain correlations across time. In risk attitudes, we found slightly higher correlations across time, both in the monetary and medical domains (Spearman’s ρ = 0.51 and ρ = 0.39, respectively; both *p* < 0.001; Fig 8, top). In contrast, in ambiguity, we found slightly lower correlations across time in the monetary and medical domains (Spearman’s ρ = 0.18 and ρ = 0.27; both *p* < 0.001, Fig. 8, bottom).

## Discussion

Many of the decisions we make have potential outcomes that are qualitative rather than quantitative. Using a novel experimental paradigm, we compared individual risk and ambiguity attitudes in a decision-making task involving both quantitative monetary outcomes and qualitative medical outcomes. Consistent with previous literature, we found that participants were generally risk and ambiguity-averse in monetary decisions.

A new contribution we make is the finding that participants are also averse to ambiguity with qualitative medical decisions. Whether participants are similarly averse to risk with medical outcomes is difficult to assess. It requires showing that when presented with options with equal expected values, participants favor certain over risky ones. However, by design, there is no objective way to estimate the expected value of qualitative outcomes. Nevertheless, an insight into risk preferences in the medical domain can be gained by comparing uncertainty attitudes across domains.

Previous literature investigated the stability of uncertainty attitudes across domains, offering no conclusive consensus on their persistence. In risk, despite having substantial domain-specificity, there is evidence for a cross-domain risk-taking disposition (Harrison et al., 2020; Highhouse et al., 2017; Prosser & Wittenberg, 2007; Vlaev et al., 2010). Research on ambiguity has been less extensive and has similarly yielded inconclusive results (Li et al., 2018; Voorhoeve et al., 2016). We extend this literature to the stability of attitudes across quantitative and qualitative outcomes, finding significant correlations in both ambiguity and risk attitudes across the monetary and medical domains. Whether the low-to-moderate correlations we identify should be considered substantial, is largely a matter of interpretation. However, a unique feature our paradigm offers in assessing these correlations is the comparison of within-domain and across-domain attitude stability.

Our findings reveal an intriguing pattern. In our data, risk attitudes were more consistent over time than they were consistent across domains. Conversely, attitudes towards ambiguity show less stability over time than across various domains. These findings correspond with previous literature which characterized, indirectly, risk attitudes as a more trait-like construct, and ambiguity attitudes as more state-like. Risk attitudes have been associated with the structure of specific brain areas, including the posterior parietal cortex (Gilaie-Dotan et al., 2014; Grubb et al., 2016), amygdala (Jung et al., 2018), and cerebellum (Quan et al., 2022), supporting the characterization of risk attitude as a stable trait-like construct. Ambiguity, in contrast, is unstable within individuals (Dimmock et al., 2016), changes when observed by peers (Curley et al., 1986; Trautmann et al., 2008), and relates to physiological (FeldmanHall et al., 2016; Jiryis et al., 2022; Katsaros & Nicolaidis, 2012; Konova et al., 2020) and emotional states (Rubaltelli et al., 2010), supporting its view as a more state-like construct.

Our study focused on uncertainty with probabilities in a medium range (25%−75%) and outcomes that were framed as gains, and both probabilities and outcomes were stated with every choice. Previous studies have highlighted the role of memory in uncertainty attitudes and how attitudes may change when involving small or very small probabilities (Reyna, 2004, 2008). Furthermore, previous research has shown that uncertainty attitudes expressed in gains are largely independent of uncertainty attitudes for outcomes framed as losses (Attema et al., 2018; Tymula et al., 2013). Future studies may expand our experimental framework to include a wider range of probabilities as well as outcomes framed as losses.

When fitting several models to behavior we found that a model that took into account participants’ explicit ratings of each outcome did not fit the data very well. This finding corresponds with previous literature on framing effects, which shows that decision-makers’ uncertainty preferences may heavily depend on their elicitation method, to the extent of preferences even being reversed in a framing of explicit desirability statement compared to actual decisions (Lichtenstein & Slovic, 1971). In contrast, a model that did fit the data well was based on the ordinal value of items. Surprisingly, this model was successful not only in the medical but also in the monetary domain. This result suggests that participants judged a given outcome not by its actual cardinal value, but rather by its rank ordering in the current choice set. This finding contributes to a broader literature that highlights the effects of choice sets on decisions (Redelmeier & Shafir, 1995). The implied ability to shape decisions by the mere configuration of a choice set (Grubb et al., 2023) is both concerning and promising, highlighting the potential to improve decision-making environments (Bovens, 2009; Thaler, 2009), in particular with the availability of a computational model (Dan & Loewenstein, 2019).

In conclusion, our study demonstrates that risk and ambiguity attitudes are broadly generalizable from quantitative to qualitative outcomes. Our modeling approach presents an economic decision-making framework which provides a quantitative estimation of subjective values in contexts implicating categorical ordered outcomes. This approach can be applied in diverse contexts, including investigations of the neural representation of qualitative outcomes or how individual differences, such as clinical symptoms, affect the subjective value of qualitative outcomes

## Data Availability

All data generated or analyzed during this study are included in this published article and its supplementary information files.
